# Severity of Post-Operative Pain after Instrumentation of Root Canals by XP-Endo and SAF Full Sequences Compared to Manual Instrumentation: A Randomized Clinical Trial

**DOI:** 10.3390/jcm11237251

**Published:** 2022-12-06

**Authors:** Ajinkya M. Pawar, Anuj Bhardwaj, Alessio Zanza, Dian Agustin Wahjuningrum, Suraj Arora, Alexander Maniangat Luke, Mohmed Isaqali Karobari, Rodolfo Reda, Luca Testarelli

**Affiliations:** 1 Department of Conservative Dentistry and Endodontics, Nair Hospital Dental College, Mumbai 400008, India; 2 Department of Conservative Dentistry and Endodontics, College of Dental Sciences & Hospital, Rau, Indore 453331, India; 3 Department of Oral and Maxillo-Facial Sciences, Sapienza University of Rome, Via Caserta 06, 00161 Rome, Italy; 4 Department of Conservative Dentistry, Faculty of Dental Medicine, Universitas Airlangga, Surabaya 60132, Indonesia; 5 Department of Restorative Dental Sciences, King Khalid University, Abha 61421, Saudi Arabia; 6 Department of Surgical Sciences, College of Dentistry, Ajman University, Al-Jurf, Ajman 346, United Arab Emirates; 7 Centre of Medical and Bio-Allied Health Sciences Research, Ajman University, Al-Jurf, Ajman 346, United Arab Emirates; 8 Department of Restorative Dentistry & Endodontics, Faculty of Dentistry, University of Puthisastra, Phnom Penh 12211, Cambodia; 9 Conservative Dentistry & Endodontics, Saveetha Dental College & Hospitals, Saveetha Institute of Medical and Technical Sciences University, Chennai 600077, India

**Keywords:** post-operative pain, endodontic instrumentation, SAF system, XPS system, K-files

## Abstract

This investigation aimed to examine the post-operative pain experienced following single-visit root canal treatment using the XP-endo shaper sequence (XPS), full-sequence self-adjusting file (SAF), and manual K-files (HKF). A randomized equivalence parallel design, double-blinded clinical study was conducted on 120 patients with symptomatic irreversible pulpitis, with or without clinical signs of apical periodontitis. Only teeth with fully formed roots and no periapical lesions were incorporated in the study. Patients were apportioned to one of three groups (*n* = 40) randomly: Group 1—XPS, Group 2—SAF, and Group 3—HKF. Pre- and post-instrumentation pain was rated utilizing Visual Analog Scale (VAS) with a spectrum of 0–100 mm. The descriptive statistics and one-way ANOVA with 95% confidence intervals were used for statistical analysis. The mean VAS scores before instrumentation were consistent in all three groups. At 6, 24, 48, and 72 h, patients with root canals instrumented by SAF had the lowest post-instrumentation mean VAS score, followed by XPS. For all time intervals, the patients in the HKF group had the highest VAS score. The full-sequence SAF instrumentation resulted in less post-operative pain than the XP-endo plus protocol, while manual instrumentation with K-files resulted in the highest post-operative pain.

## 1. Introduction

The term “post-endodontic pain” describes any uncomfortable feeling or tenderness that transpire after an endodontic treatment. The prevalence of such pain has been estimated between 25% and 40% of cases. Furthermore, regardless of pulp status or peri-radicular condition, the frequency of post-operative pain recorded in patients is approximately 50–60% [1].

Debris expulsion from the apical foramen in the course of the biomechanical preparation of the root canal has been associated with post-operative pain [2]. Such debris may include microorganisms, irrigating solutions, infected dentin particles and pulpal tissue remnants, and their extrusion could depend on different factors; among those, the instrumentation strategies and irrigation play a crucial role [3]. It is believed that the number of extruded debris is responsible for the incidence and may increase the frequency of post-operative pain [4]. The intensity of pain may vary depending on the degree of periapical tissue injury. The method of evaluation of the level of pain endured by patients after root canal treatment using various root canal instrumentation methods has been well established [5,6].

It has been observed that different file systems, which use different preparation methods, such as rotary or reciprocating files, may result in varying levels of post-operative pain. The tip diameter, file designs, taper, and preparation method all can have an effect on the pain response [7].

When slender root canals with an annular cross-section are considered, rotating and reciprocating files may adequately clean the root canal and its walls. In the case of oval root canals, on the other hand, rotating files leave much to be desired [8]. This constraint prompted the development of new file systems, such as the self-adjusting file (SAF; ReDent Nova, Ra’anana, Israel) and the XP-endo Shaper and Finisher (XPS and XPF; FKG Dentaire, La Chaux-de-Fonds, Switzerland), both of which claim to be adaptive root canal instrumentation methods that can also effectively clean irregularly shaped canals [9].

The SAF was developed primarily to solve the issue of root canals with non-round cross-sections. It attempts to address diverse pitfalls of rotary and reciprocating files when manipulated in root canals with variable cross-sections. The SAF is a NiTi instrument, which has the shape of a hollow tube made of thin-walled lattice. When inserted into the canal, it can be squeezed from its 1.5 mm diameter to the proportions of a #20 K-file [10,11]. The file is used with a special hand-piece head, which turns the rotation into transline in-and-out vibrations with a 0.4 mm amplitude. The extreme compressibility and elasticity allow the SAF to alter itself to the cross-section of any given root canal, including oval canals. The SAF is employed with uninterrupted irrigation, which is implemented through the hollow file, using a peristaltic pump [10,11].

Previously, the manufacturer instructions indicated the need to prepare a glide path for the SAF using a manual #20 K-file. This instruction recently changed to recommend motorized glide path preparation using a pre-SAF orifice shaper (#40/0.10), pre SAF 1 (#15/0.02) in cases of narrow/calcified canals, and pre SAF 2 (#20/0.04), which should allow a 1.5 mm SAF to be manually inserted to working length prior to its activation. This new sequence of instrumentation has been termed “full-sequence SAF” [12].

During instrumentation, the newly developed XP-endo Shaper plus guidelines involves three junctures: (i) K-files (#10 and #15) are utilized to generate a glide path. Thereafter, (ii) the XP-endo Shaper and (iii) XP-endo Finisher are utilized. These two instruments are spun at 800 RPM with pecking movement patterns to reach working length [13].

The XP-endo Shaper file (#30/0.01) consists of Max wire NiTi alloy and has a zigzag shape. The tip terminus has six cutting blades that flow smoothly to the shaft and features an inactive, bullet-like apical section. The instrument is impacted by body temperature, which enhances it to an austenitic juncture, allowing it to operate in a snake-like manner. It is first employed as a piercing instrument in the root canal, and then 15 pecks are added to the working length after the working length is established. After XP-endo Shaper instrumentation, the resultant root canal size is #30/0.04 [14]. The semi-circular whip-shape of the XP-endo Finisher file (#25/0.00) is originally founded as a compatible instrument for use following root canal shaping with any file system. This file is designed to clean up the root canal system’s complicated morphologies and hard-to-reach areas [15].

The apical outflow of debris caused by the manufacturers’ proposed protocol for the two adaptive file systems was compared to conventional root canal instrumentation with K-files in vitro [13]. It was found that each juncture of these two procedures makes its own augmentation to the apical expulsion of debris; thus, the complete sequence of instrumentation should be considered when evaluating the instrument’s contribution to apically extruded debris [13]. The present study was designed to clinically evaluate the post-operative pain that occurs following the use of these two adaptive file systems and compares it to the pain that occurs following traditional manual K-file instrumentation of the root canals.

## 2. Materials and Methods

### 2.1. Sample Allocation and Ethical Approval

The reflected randomized clinical trial adopted ethical norms including the World Medical Association Declaration of Helsinki (Carlson, Boyd, and Webb, 2004) and the PRIRATE 2020 standards [16]. The study was a prospective, parallel group, equivalence, double-blinded, single center, randomized clinical trial. It was approved by the Madhya Pradesh Medical Science University’s Institutional Ethical Committee (CDSH/IEC/2019-2020/006) and registered also with Clinical Trials Registry-India (CTRI/2020/02/023339). This in vivo study was executed on 120 patients, which were randomly designated to 3 groups. Sample size was determined using the mean and standard deviation values from Emara et al. [17]. The power was set at 80%, while the alpha-level was set to 0.05. Approximately 37 subjects/samples per group need to be taken in the present study. To compensate for potential losses over follow-ups, this number was increased to 40 patients for each group. The G*Power: Statistical Power Analyses and Sample size Calculation Software, version 3.1.9.3, was used to calculate the sample size (Heinrich Heine University, Dusseldorf, Germany).

### 2.2. Patient Selection

A total of 200 consecutive patients aged between 18 and 65 years were screened between 14 September 2020 and 17 March 2021 for the selection criteria. Patients diagnosed with symptomatic irreversible pulpitis with or without clinical signs of apical periodontitis, with fully formed mature roots and an absence of periapical lesion, with respect to maxillary and mandibular first molars, were included in this study. Pulp vitality was confirmed using a cold test (Endo-Ice; Hygienic, Akron, OH, USA), and an electric pulp test. A preoperative periapical radiovisiograph (RVG; Vatech India, New Delhi, India) was also taken for the tooth in question.

Exclusion criteria included patients with non-vital teeth (negative thermal stimulation with Endo-Ice validated with nonappearance of bleeding during access cavity preparation; see below); cases of previous endodontic retreatment, second and third molars, and intentional root canal treatment were also excluded. Further exclusion criteria were cases with periapical radiolucent lesion, root resorption, immature/open apex, root caries, and gross decay that were considered non-restorable as well as teeth with mobility and complex anatomy. Also excluded were patients who declined to cooperate in the study, those who had taken medication in the 12 h before the operation, such as analgesics or nonsteroidal or steroidal anti-inflammatory drugs, pregnant and breastfeeding patients, patients with any uncontrolled systemic disease, and patients younger than 18 or older than 65 years. Thus, in total, 120 patients were incorporated and randomized to three study groups.

### 2.3. Randomization, Allocation Concealment, and Blinding

After being versed about the trial and providing written consent, the patients were randomly assigned to three groups using a computer-generated simple randomization method (www.random.org, accessed on 25 March 2021): Group 1 = XPS, Group 2 = SAF, and Group 3 = HKF. The staff nurse enclosed the randomized order of instrumentation procedures in opaque separate envelopes, which were later opened by the operator on starting endodontic treatment. All procedures were performed by two skilled operators (endodontists). Before commencement of this trial, a standardization protocol was established by the operators. The protocol for each instrumentation system was calibrated for the operators. The operators could not be blinded, as each instrumentation system had to be implemented in its unique way. The patients and outcome assessor were blinded to the groups of the study.

**Figure 1:**
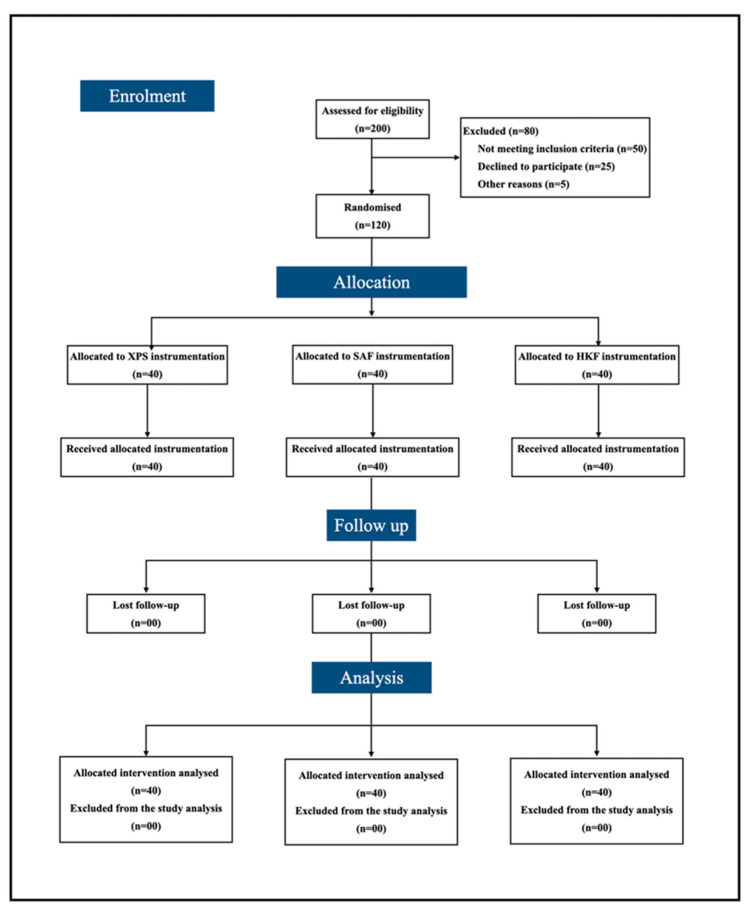
COSORT 2010 flowchart.

Case history sheets were filled by the treating clinicians, covering standard parameters such as age, gender, medical history, the tooth to be treated and vitality status of the tooth. Pre-instrumentation pain level was recorded using a Visual Analog Scale (VAS ranging from 0–100 mm), the use of which was first explained to the patient and used for both preoperative and postoperative pain assessment. Patients with a preoperative pain level of a minimum of 50 (>50) according to the VAS were included. The randomization and allocation of participants to different groups with analysis is explained in detail in Figure 1.

### 2.4. Treatment Protocol

Local anesthesia (2% lidocaine 1:80,000 epinephrine) (Lidayn, Dr. Dentaids, Kolkata, India) was administered. Following rubber dam application, an access opening was prepared. The full procedure was executed under a dental operating microscope (DOM 3000 B-Silver; Semorr CricDental, Mumbai, India). Upon exposure of the pulp chamber, the presence of hemorrhage was noticed (indicating pulp vitality); only teeth showing such hemorrhage were included in this trial. Cases with no such hemorrhage were considered non-vital and were debarred from the study, and another patient was assigned. Working length was established using an electronic apex locator (Propex Pixi, Dentsply India, Mumbai, India) and was set at 0.5 mm short of the “apex” indicator length. Cases in which no apex locator reading was possible were debarred from the evaluation and another suitable tooth/patient was assigned.

### 2.5. Root Canal Instrumentation

#### 2.5.1. XPS plus Sequence

##### Stage 1: Glide Path Initiation

The instrumentation of root canals was done at this stage using a #15 K-file effectively until reaching the working length to establish a glide path, as per the manufacturer’s directives. For repeated irrigation, pre- and post-instrumentation, a syringe with a 30-G needle (Neoendo; Orikam, Mumbai, India) was used with 2 mL of 3% sodium hypochlorite (Prime Dental Pvt. Ltd., Mumbai, India).

##### Stage 2: XP-Endo Shaper Shaping

The XP-endo Shaper (XPS) file was then used to prepare the root canal while in its austenite form, which was induced by body temperature. The XPS file was placed into the root canal in a passive manner to the extent of resistance. Once resistance was hit, the file was then drawn out, and the endomotor (X-Smart Plus; Dentsply India) was set in motion. The file was powered at 800 rpm and 1 Ncm torque. The file was utilized with long gentle pecks 4–5 times on the way to attain the working length. Once the working length was accomplished, the file was retracted and cleaned, apical patency was verified with a 15 K-file, the root canal was flooded with 4 mL 3% sodium hypochlorite, and the file was used moreover for an additional 15 pecking strokes up to the working length with intermittent flooding of the root canals with a total of 12 mL (4 mL per 5 strokes).

##### Stage 3: XP-Endo Finisher Finishing

The root canal was flooded with 3% sodium hypochlorite, the XP-endo finisher (XPF) was retrieved from the differentiating plastic cannula, and the rubber reference stopper was specified to working length during this phase. The same endomotor (X-Smart Plus) was used to operate the file at 800 rpm and 1 Ncm. The XPF was inserted to WL, the canal was loaded with 2 mL 3% sodium hypochlorite, and the instrument was driven in the canal for 60 s. Soft 7 to 8 mm in-and-out movements to working length were employed, and subsequent to this, a final flush of 2 mL 3% sodium hypochlorite was achieved.

#### 2.5.2. SAF Full Sequence

##### Stage 1: Glide Path Initiation

A glide path was prepared using a torque-managed endomotor (X-Smart Plus) according to the manufacturer’s recommendations. The Pre-SAF OS (#40/0.10) was executed as an orifice shaper in the coronal 3 mm of the root canal at 600 rpm and 1.5 Ncm, supplemented by the Pre-SAF 1 (#15/0.02) and Pre-SAF 2 (#20/0.04). Both were applied with a pecking motion using 2–3 gentle movements until the working length was reached. With each instrument, the canals were irrigated with 2 mL sodium hypochlorite using a syringe and a 30-G needle (Neoendo), for a total of 6 mL. A 10 K-file was used to verify the root canal’s patency at the assertion of the glide path preparation, and subsequent to this, a final flush with 2 mL sodium hypochlorite was achieved.

##### Stage 2: Cleaning and Shaping

To test and verify the glide path, 1.5 mm SAF was manually and gently inserted into the canal up to the working length. Employing a pre-programmed EndoStation (ReDent Nova), the root canal instrumentation was accomplished for four minutes in each canal. It was used at a frequency of 5000 vibrations per minute and an amplitude of 0.4 mm. While the file reached working length, a pecking motion was implemented. With each pecking motion, the outbound component of each stroke was lengthy enough to allow the file to shift its circular location. Irrigation was achieved with 3% sodium hypochlorite, which was delivered along the hollow file by an integrated peristaltic pump (ReDent Nova). With a cumulative irrigant quantity of 16 mL, the flow rate was set at 4 mL/min.

#### 2.5.3. Manual Instrumentation

Hand instrumentation was executed by the step-back technique with stainless steel K-files. The sequence of instruments was #10/0.02, #15/0.02, #20/0.02, #25/0.02, #30/0.02, and #35/0.02 hand K-files (Mani Inc., Tokyo, Japan). After initial coronal flaring with Gates-Glidden burs #2 and #3 (Mani), the canal was gradually enlarged to a final apical size of #35. Root canal preparation was brought out using an in-and-out filing motion. The preparation was completed using the step-back technique with recapitulation. Irrigation was performed between instruments using 3 mL 3% sodium hypochlorite, delivered with a syringe and needle, as in the other groups. A total of 18 mL of irrigant was used. The patency of the root canal was confirmed with a 10 K-file at the end of the preparation, followed by a final flush with 4 mL sodium hypochlorite using a syringe and needle.

A cumulative of 22 mL of irrigant was used in all the groups to standardize the volume of sodium hypochlorite. Additionally, for effective smear-layer removal, all root canals were irrigated with 3 mL of 17% EDTA solution (Prevest Denpro Limited, Jammu, India) through a syringe fitted with a 30-G needle (Neoendo; Orikam, Mumbai, India) placed 2 mm short of the WL. This solution was activated ultrasonically (Irrisonic tip; Helse, Santa Rosa do Viterbo, SP, Brazil) at a minimum power setting (10%), inserted into the canal 1 mm short of the WL. The canals were then finally rinsed with 10 mL of saline solution.

### 2.6. Root Canal Obturation

**Figure 2:**
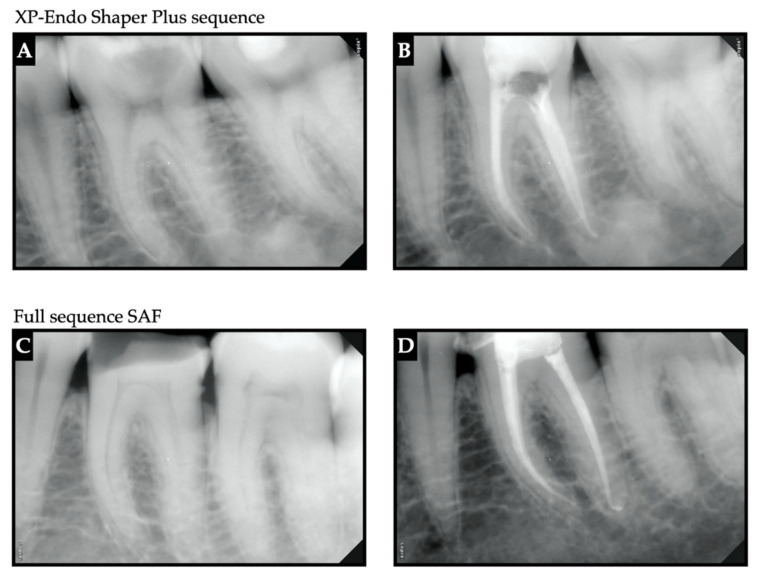
Representative radiovisiographs (RVGs) of pre-operative and post-obturation for XP-endo shaper plus sequence (mandibular second molar) (**A**,**B**) and full-sequence SAF (mandibular first molar) (**C**,**D**).

The canals were dried by paper points following instrumentation and subsequent irrigation. Teeth were obturated using a resin-based sealer (AH plus, Dentsply, Konstanz, Germany) and a gutta-percha master cone (35/04 for SAF, 30/04 for XPS, and 35/02 for HKF) at the first initial visit. For all groups, accessory cones were added using the lateral compaction technique. Teeth were then sealed with a sterile dry cotton pellet and hydraulic temporary restorative material (MD-Temp Plus, Chungcheongbukdo, South Korea). Patients were scheduled for a permanent restoration within 10–15 days (Figure 2).

Following the endodontic treatment procedure, all patients were instructed to take analgesics (400 mg ibuprofen) at a therapeutic dose of 1 tablet every 6 h if unbearable pain occurred.

### 2.7. Outcome Assessment

The principal outcome was post-operative pain severity, appraised using a visual analogue scale (VAS) at 6, 24, 48, and 72 h. All patients were given a VAS (ranging from 0 to 100 mm) to take with them so that they could aid the outcome assessor with pain level assessment while filling out the post-operative questionnaire. The intensity of pain (primary outcome) was classified into four categories: no pain (level A, 0–24 mm), mild pain (level B, 25–49 mm), moderate pain (level C, 50–74 mm), and severe pain (level D, 75–100 mm). The scale was explained to the patients both before treatment, when assessing the preoperative pain level and again before leaving the clinic. It was done visually, verbally, and numerically to facilitate its use, and the patients were trained on how to fill out the VAS score. They were contacted by the outcome assessor by telephone at a suitable time, approximately 6 h, 24 h, 48 h, and 72 h after the treatment to record VAS scores for post-operative pain.

### 2.8. Statistical Analysis

Data obtained were entered and sorted in Microsoft Excel (2017), and statistical analysis was executed using Statistical Package for Social Sciences (SPSS) software 21.0 version. The baseline characteristics of each group (age, gender, tooth position, VAS scores, and dosage of analgesics used) were compared. The normality of the data was checked with the Shapiro–Wilk normal distribution test (*p* > 0.05). As the data were normally distributed, parametric tests were implemented. Descriptive statistics were performed to determine the mean VAS scores in all the groups at different time intervals. A paired sample t-test was used to assess the change in VAS score from pre- to post-instrumentation. One-way ANOVA with post hoc Tukey’s test was used to find a significant inter-group comparison of post-instrumentation VAS scores at different time intervals. The reliability between the two independent operators was assessed using the Cohen’s kappa, considering a statistically significant *p* value of less than 0.05 at 95% confidence intervals.

## 3. Results

The research included 40 patients in each group: Group 1—XPS, Group 2—SAF, and Group 3—HKF. The demographic details of the patients were: the mean age of the patients was 32.53 (±6.612) years, 30.19 (±8.256) years, and 28.88 (±5.194) years in Group 1, Group 2, and Group 3, respectively (Table 1). When gender distribution was assessed, there was no difference between the groups (Table 1).

The primary outcome was evaluated using VAS score (0–100 mm) for all three groups. The descriptive statistics were performed for pre-operative and post-operative VAS score following the endodontic instrumentation using a particular file system involved. The mean pre-instrumentation VAS score was almost identical in all three groups (Table 2).

At 6 h post instrumentation, the mean VAS score was found to be the lowest for the SAF group (31.39 ± 7.22 mm), followed by the XPS group (41.75 ± 7.81 mm) and then with HKF (50.55 ± 4.96 mm) (Table 2).

Similarly, at 24 h, 48 h and 72 h post-instrumentation, the mean VAS score in the SAF group was 26.15 (±5.62), 16.17 (±6.42) and 12.57 (±2.64), respectively. For the XPS Group, it was 29.62 (±5.47), 21.3 (±3.59) and 15.82 (±2.47) and for the HKF group was 38.4 (±5.52), 31.15 (±5.17) and 21.95 (±2.70) at 24 h, 48 h and 72 h, respectively (Table 2).

The pre- and post-treatment comparison showed statistically notable differences (*t* test, *p* value = 0.000) in all three groups and showed an overall lesser VAS score with passing time. The intergroup comparison was accomplished using One-way ANOVA and Post hoc Tukey’s test to determine the significant differences between the three groups. There was no significant difference in pre-operative pain between the three groups. At 6 h, there were significant differences in post-operative pain between SAF and XPS, SPS and HKF, and XPS and HKF. Similarly, there were significant differences in post-operative pain at 24 h, 48 h and 72 h (*p* value < 0.05). This suggested that the SAF group had the lowest mean VAS score at each time point, followed by the XPS group and then the HKF group (Table 3 and Table 4).

There was a non-statistically significant difference in the frequency of analgesic medication intake between the three groups studied (*p* = 0.149). The medication if taken was only in the first 24 h after the treatment. Ibuprofen was used by eight patients in the manual group, four in the XPS group and three patients in SAF group (after the completion of the treatment until 24 h). The Cohen’s kappa coefficient between the two clinicians was 0.85, indicating acceptable operator reliability.

## 4. Discussion

Post-operative pain succeeding root canal instrumentation with three divergent endodontic file systems was evaluated in this randomized clinical trial. It has been previously established that the SAF extruded less debris and caused less post-operative pain when used for instrumentation compared to rotary and reciprocating NiTi files [13,18,19,20].

The instrument systems used in this study were two adaptive file systems—the XPS and SAF systems, each used with a full sequence of instruments as recommended by their manufacturers and the traditional manual instrumentation using K-files. The file systems were compared to each other as a whole system, each used according to its manufacturer instructions. Thus, there was no attempt to artificially equalize the amount of irrigant used or other parameters such as number of instruments, or the time required to complete the procedure. Single-visit endodontic treatment was chosen in the present study to keep the treatment protocol straightforward and transparent and to reduce the possibility of intracanal medication discrepancies that might affect post-operative pain.

The patients in all groups presented with irreversible pulpitis, which explains the high pre-treatment visual analogue scale (VAS) scores for pain. In all groups, the pain decreased following treatment and continued to gradually reduce with time. Similar results of gradually diminishing pain have been noted in various studies [21,22,23] as well as in two systematic reviews [24,25].

In the current analysis, post-instrumentation pain was lower in the full-sequence SAF cluster at all time points compared with the other two groups (*p* value = 0.000). Manual instrumentation with K-files resulted in the highest post-operative discomfort. This finding is in agreement with previous studies that have reported that the post-operative pain level was higher when hand files were used compared to the use of multi-file rotary file systems of tapers ranging from 0.04–0.06. Single-file reciprocal systems were also reported to have higher post-operative pain levels than multi-file rotary file systems [26,27,28].

It is commonly assumed that post-treatment pain and discomfort is related to apical extrusion of debris [3,12,19,29]. Therefore, it is of interest to point out that a recent in vitro study on the consequence of the instrumentation method on the amount of apically extruded debris showed results that may explain the present clinical results [14]. This study reported that manual instrumentation with K-files resulted in a greater quantity of apically expelled debris than instrumentation with an XP-endo Shaper. Another recent in vitro study is more directly relevant to the present one; Pawar et al. [13] studied the number of debris extruded by the full-sequence of SAF and the XP-endo Shaper Plus sequence. The protocols used in vitro in that study were similar to the ones used clinically in the current study. The total amount of debris expelled during instrumentation with the XP-endo Shaper plus, in that study, was almost twice that extruded by the full sequence SAF instrumentation. It is of interest to note that this recent study reported that each stage of both protocols contributed to the gross number of debris extruded by the newer approach [13]. These recent in vitro results may elucidate the results of the reported clinical study and may also support the concept that the magnitude of post-operative pain may be coupled to the quantity of debris that is apically expelled by a given procedure [12,13,14,19].

The manual use of K-files was previously reported to (1) cause greater extrusion of debris and (2) result in more post-operative pain when compared to multi-file motorized file systems [12,14,19]. This has been attributed to the piston-like action of the files when attempting to get them to working length. Both motorized methods that were used in the present study had the potential to push debris beyond the apex. Both systems are used with repeated pecking motions to working length and both have potential difficulty in accurately controlling the depth of insertion [13]. A possible explanation for the difference between the two motorized systems in both the in vitro extrusion of debris and the amount of clinical post-operative pain may be in (1) the shape and dimensions of the tip of the file and (2) the irrigation method used.

The shape of the tip of the file and its potential to act as a piston are different in the two motorized files used in the current study. The XP-endo Shaper’s circular directing tip may serve as a plunger, thrusting and expelling debris via the apical foramen as it glides in and out of apical portion of the canal. The tip of the SAF, on the other hand, implies a rectangular cross-section of 0.12 × 0.16 mm when adequately squeezed into the circular apical portion of the canal corresponding to a #20 k-file [10,29]. This rectangular component changes its round position on a continual basis, preserving the canal’s round cross-section at the apex. The rectangular tip of the file does not perform as a piston while advancing in and out of the round apical region of the canal because of 40% of the cross-section of the canal being available for return flow [10,29]. The polarity in debris extrusion between the two file systems may also be partly explained by this variance [13].

The XPS sequence requires periodic manual irrigation (syringe and needle) amid file execution, that is another dividing line betwixt the two file systems. The SAF technique, in contrast, allows for continuous irrigation administered into the root canal via the hollow file for the whole of the procedure. It is indeed realizable that uninterrupted irrigation, which takes debris coronally and out of the canal, benefits in avoiding debris collection, decreasing the likelihood of debris being pushed through the apical foramen [13]. These differences in the apical extrusion of debris may explain the differences in post-operative pain that were found in the present study.

Previous studies have suggested that SAF [20,30] and XP-endo shaper [27,28] induced decreased post-operative pain when applied alone for root canal instrumentation. When the XP-endo finisher [31] was used as supplemental instrumentation, it caused less post-operative pain. These studies have been carried out in comparison with rotary or reciprocating files. However, no studies were done so far on the impact of the full XP-endo Shaper Plus and full-sequence SAF procedure on post-operative pain severity.

**Figure 3:**
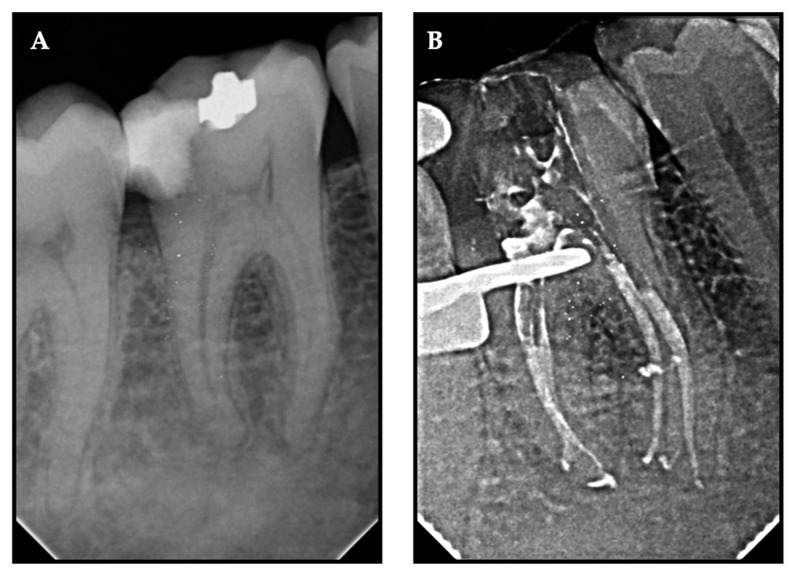
Representative radiovisiographs (RVGs) of (**A**) pre-operative and (**B**) post-obturation X-ray exhibiting sealer extrusion.

To fill all of the imperfections in the root canal system, endodontic sealers should have the ideal flow properties. Insufficient flowability may limit appropriate filling, while increased flow rate may raise the possibility of extrusion. Filling material that flows past the apex may cause more pain or may contact significant anatomical landmarks [32]. For the SAF and manual groups were not associated with sealer extrusion whereas in the XPS group exhibited sealer extrusion in 9 cases (Figure 3). However, no flare up was reported by any patient, which is in accordance with Graunaite et al. [33]. One could infer that sealers’ cytotoxic effects might affect how painful treatment feels. However, our results revealed that pain persisted with or without sealer extrusion. Consequently, our clinical findings did not support any possible negative consequences discovered in vitro that were reported by Rodriguez-Lozano et al. recently [34].

It is commonly acknowledged that the success rate and post-obturation pain will depend on the operator’s high level of skill. Law and associates revealed that it was challenging to determine how clinical experience affected post-obturation discomfort [35]. According to Glennon et al. [36], there is no discernible difference in the post-operative discomfort felt by patients who receive treatment from different skilled practitioners. This was proven again by Wong et al. [37]. In the current study, both the clinicians were experienced with more than 10 years’ experience in endodontics, and the values were in substantial agreement.

The subjective assessment of pain is one of the primary issues in pain research. Any decision to take analgesics will therefore be based on this subjective judgement. Ibuprofen was preferred as the postoperative pain medication in this investigation because nonsteroidal anti-inflammatory drugs have been proposed as first-choice medication for postoperative pain management after endodontic procedures. Furthermore, ibuprofen has been included in several studies examining the effect of various techniques and medications on pain relief following endodontic treatment [7]. The uptake of medication was higher by patients enrolled in the manual group, but the groups did not differ significantly.

### Limitations

It should be acknowledged that since comparisons were made between three processes in the current study, it was deemed inappropriate and inconsequential to make an effort to level out variables like the quantity of irrigant used, the amount of time needed, or the number of instruments used in any of the groups. Another potential limitation of the study is related to the use of various tooth types. Since discomfort in the apical region of even one root is likely to be mistaken for pain in the treated tooth, a three-rooted molar may be more likely to feel pain after treatment than a single-rooted tooth.

## 5. Conclusions

The full sequence SAF instrumentation resulted in less post-operative pain than the XP-endo Plus protocol, while manual instrumentation with K-files resulted in the highest post-operative pain.

## Figures and Tables

**Table 1:** Demographic details and teeth treated.

	XPS	SAF	HKF	*p* Value
Age (Mean ± SD)	32.53 (±6.612)	30.19 (±8.256)	28.88 (±5.194)	0.479
Male	20	22	19	0.531
Female	20	18	21	
Maxillary Molars	21	18	20	0.296
Mandibular Molars	19	22	20	

*p* Values were >0.05 exhibiting statistically insignificant difference in the distribution of data.

**Table 2:** Pre-operative and post-operative pain. Mean VAS scores (±SD).

		Mean (±SD)		Mean Difference	*p* Value
SAF	Pre op pain	58.4750	4.21224	27.08000	0.000
	6 h Post op pain	31.3950	7.22786		
	Pre op pain	58.4750	4.21224	32.32500	0.000
	24 h Post op pain	26.1500	5.62996		
	Pre op pain	58.4750	4.21224	42.30000	0.000
	48 h Post op pain	16.1750	6.42067		
	Pre op pain	58.4750	4.21224	45.90000	0.000
	72 h Post op pain	12.5750	2.64950		
XPS	Pre op pain	58.7500	4.45346	17.00000	0.000
	6 h Post op pain	41.7500	7.81763		
	Pre op pain	58.7500	4.45346	29.12500	0.000
	24 h Post op pain	29.6250	5.47576		
	Pre op pain	58.7500	4.45346	37.45000	0.000
	48 h Post op pain	21.3000	3.59629		
	Pre op pain	58.7500	4.45346	42.92500	0.000
	72 h Post op pain	15.8250	2.47953		
HFK	Pre op pain	59.4000	4.33708	8.85000	0.000
	6 h Post op pain	50.5500	4.96630		
	Pre op pain	59.4000	4.33708	21.00000	0.000
	24 h Post op pain	38.4000	5.52755		
	Pre op pain	59.4000	4.33708	28.25000	0.000
	48 h Post op pain	31.1500	5.17662		
	Pre op pain	59.4000	4.33708	37.45000	0.000
	72 h Post op pain	21.9500	2.70754		

*p* Values < 0.05 were considered as statistically significant difference.

**Table 3:** Intergroup post-instrumentation comparison between three groups at different time interval.

		Sum of Squares	df	Mean Square	F	Sig.
Pre op pain	Between Groups	18.050	2	9.025	0.480	0.620
6 h Post op pain	Between Groups	7354.401	2	3677.200	79.927	0.000
24 h Post op pain	Between Groups	3188.517	2	1594.258	51.855	0.000
48 h Post op pain	Between Groups	4633.850	2	2316.925	85.859	0.000
72 h Post op pain	Between Groups	1812.917	2	906.458	132.661	0.000

**Table 4:** Multiple pairwise comparisons using Tukey HSD.

Dependent Variable	Group	Group	Mean Difference	*p* Value
Pre op pain	SAF	XPS	−0.27500	0.957
		HKF	−0.92500	0.607
	XPS	HKF	−0.65000	0.781
6 h Post op pain	SAF ^1^	XPS ^2^	−10.35500 *	0.000
		HKF ^3^	−19.15500 *	0.000
	XPS ^2^	HKF ^3^	−8.80000 *	0.000
24 h Post op pain	SAF ^1^	XPS ^2^	−3.47500 *	0.016
		HKF ^3^	−12.25000 *	0.000
	XPS ^2^	HKF ^3^	−8.77500 *	0.000
48 h Post op pain	SAF ^1^	XPS ^2^	−5.12500 *	0.000
		HKF ^3^	−14.97500 *	0.000
	XPS ^2^	HKF ^3^	−9.85000 *	0.000
72 h Post op pain	SAF ^1^	XPS ^2^	−3.25000 *	0.000
		HKF ^3^	−9.37500 *	0.000
	XPS ^2^	HKF ^3^	−6.12500 *	0.000

* *p* Value < 0.05 statistically significant; ^1,2,3^ superscripts show the most effective file system in numerical sequence showing statistically significant differences.

## Data Availability

Not applicable.
